# Adverse health outcomes and health-related quality of life (HRQoL) among long-term adolescent and young adult (AYA) brain tumour survivors: results from the population-based SURVAYA study

**DOI:** 10.1007/s00520-025-09155-9

**Published:** 2025-01-14

**Authors:** Konstantinos Angoumis, Catarina S. Padilla, Mathilde C. M. Kouwenhoven, Rhodé M. Bijlsma, Suzanne E. J. Kaal, Jacqueline M. Tromp, Monique E. M. M. Bos, Tom van der Hulle, Martinus P. G. Broen, Janine Nuver, Winette T. A. van der Graaf, Sophie Pauge, Olga Husson

**Affiliations:** 1 University of Bielefeld, School of Public Health, 33615 Bielefeld, Germany; 2 Department of Medical Oncology, Netherlands Cancer Institute – Antoni van Leeuwenhoek, 1066 CX Amsterdam, the Netherlands; 3 Department of Medical Oncology, Erasmus MC Cancer Institute, Erasmus University Medical Center, 3015 GD Rotterdam, the Netherlands; 4 Department of Neurology, Cancer Center Amsterdam, Amsterdam UMC, Amsterdam University Medical Centers, Location VUmc, 1081 HV Amsterdam, the Netherlands; 5 Department of Medical Oncology, University Medical Center Utrecht, 3584 CX Utrecht, the Netherlands; 6 Department of Medical Oncology, Radboud University Medical Center, 6525 GA Nijmegen, the Netherlands; 7 Department of Medical Oncology, Amsterdam University Medical Centers, 1105 AZ Amsterdam, the Netherlands; 8 Department of Medical Oncology, Leiden University Medical Center, 2333 ZA Leiden, the Netherlands; 9 Department of Neurology, GROW‑School of Oncology and Reproduction, Maastricht University Medical Center+, 6229 HX Maastricht, the Netherlands; 10 Department of Medical Oncology, University Medical Center Groningen, 9713 GZ Groningen, the Netherlands; 11 School of Public Health, Department of Health Economics and Healthcare Management, University of Bielefeld, 33615 Bielefeld, Germany; 12 Department of Surgical Oncology, Erasmus MC Cancer Institute, Erasmus University Medical Center, 3015 GD Rotterdam, the Netherlands

**Keywords:** Survivorship, Health-related quality of life, Late effects, Neurooncology, Adverse health outcomes

## Abstract

**Purpose:**

Adolescent and young adult (AYA) malignant brain tumour (BT) survivors are at risk of adverse health outcomes, which may impact their health-related quality of life (HRQoL). This study aimed to investigate the (1) prevalence of physical and psychological adverse health outcomes, (2) the HRQoL, and (3) the association of adverse health outcomes and HRQoL among long-term AYA-BT survivors. Adverse health outcomes and HRQoL were compared to other AYA cancer (AYAC) survivors.

**Methods:**

A cross-sectional secondary data analysis of the SURVAYA study among 133 AYA-BT and 3877 AYAC survivors was conducted. Participant self-reported adverse health outcomes and HRQoL scores were analysed and compared between the two populations. Associations with HRQoL were assessed using linear regression modelling with AIC-based backward elimination.

**Results:**

AYA-BT survivors faced significant issues of fatigue (47.6%), future uncertainty (45.2%), and medical conditions like vision (34.4%), speech, taste, or smell (26.2%) impairments, cancer recurrence, and metastasis (25.4%). Neurocognitive symptoms were identified as BT-specific issues (13.6–33.6%). Compared to AYAC survivors, AYA-BT survivors reported significantly (*p* < 0.05) lower functioning scores on the role, cognitive, emotional, and social HRQoL, with cognitive (56.0%) and emotional (40.0%) domains being the most affected. Adverse health outcomes were negatively associated with HRQoL, ranging from small to large clinical relevance.

**Conclusion:**

Long-term AYA-BT survivors were identified as a highly burdened population, affected by multifaceted issues and multidimensional detriments in HRQoL years beyond their cancer diagnosis. This study highlights the necessity of long-term follow-up and a holistic, multidisciplinary approach to survivorship care to ultimately improve the quality of AYA-BT survivorship.

**Supplementary Information:**

The online version contains supplementary material available at 10.1007/s00520-025-09155-9.

## Introduction

Central nervous system cancers are a histologically complex and heterogeneous group of neoplasms, with the majority comprising malignant brain tumours (BTs) [1]. Although BTs account for a relatively small proportion of total cancer cases in Europe (1.7% in 2020) and the Netherlands (1.3% in 2020), they pose significant challenges to healthcare systems due to their complex nature and treatment requirements [2, 3]. These neoplasms represent a significant health concern for adolescents and young adults (AYAs) in the Netherlands. In 2020, the incidence rate among AYAs was 2.4 (per 100,000), and central nervous system tumours were the second leading cause of cancer-related deaths (mortality rate 1.2 per 100,000), underscoring their importance in this age group [4]. AYAs (18–39 years), in general, form a unique cancer population, characterised by increased vulnerability due to developmental challenges and physical, emotional, cognitive, and social transitions disrupted by a cancer diagnosis and its treatment [5].

BTs warrant special attention in AYAs due to high morbidity and mortality, diminished health-related quality of life (HRQoL), and associated changes in social and occupational roles [6, 7]. Although BT patients face a limited or uncertain survival prognosis compared to other types of cancer, the population of long-term AYA-BT survivors (≥ 5 years) is expanding due to medical advancements [8–10]. Multimodal and adjuvant cancer treatments put AYA-BT survivors at risk of facing disease- or treatment-related long-term and late effects across the dimensions of the biopsychosocial model, including neurocognitive impairments or fatigue [10, 11]. Long-term and late effects among AYAs are sometimes also related to their young age, such as psychological issues, including mood disorders and fear of cancer recurrence [11]. These adverse health outcomes may impact survivors’ HRQoL and may lead to diminished quality of long-term survival [10, 12–14].

Given that AYA-BT survivors represent a distinct population, further understanding of long-term physical and psychological challenges, along with their impact on HRQoL, is imperative [9, 11, 15]. To the best of our knowledge, a study examining adverse health outcomes and their association with long-term AYA-BT survivors’ HRQoL has not been conducted yet. Thus, this study aims to enhance the understanding of long-term health outcomes and HRQoL among AYA-BT survivors by (1) quantifying the prevalence of physical and psychological adverse health outcomes, (2) investigating long-term cancer survivors’ HRQoL, and (3) examining the association of adverse health outcomes and HRQoL. Additionally, with regard to objectives (1) and (2), the prevalence of adverse health outcomes and HRQoL is compared to other AYA cancer (AYAC) survivors to determine AYA-BT-specific issues.

## Methods

### Study design

A cross-sectional secondary data analysis of the SURVAYA study was conducted following the guidance of the STROBE checklist [16].

### Data set, study population, and measures

The population-based SURVAYA study (NCT05379387) aimed to investigate the prevalence, risk factors, and mechanisms of long-term adverse health outcomes of cancer and its treatment in AYA cancer survivors. In short, the study was conducted between 2019 and 2021 in the Netherlands and included individuals diagnosed with a malignant neoplasm during adolescence and young adulthood (18–39 years) 5–20 years before study participation. AYAs were treated at the Netherlands Cancer Institute (NKI) or a university medical centre in the Netherlands. Further information on the study design, recruitment process, and sample characteristics have been described elsewhere [5]. Patient-reported outcome (PRO) data were collected via the PROFILES registry, while certain sociodemographic and clinical data were retrieved from the Netherlands Cancer Registry (NCR). For this study, the population of long-term AYA-BT survivors was identified based on the NCR tumour type codes equivalent to the ICD-O-3 codes C71, C72.2–3, C75.1, and C75.3.

In total, 133 individuals were identified as AYA-BT survivors. Sociodemographic characteristics were self-reported except for age and sex, with data available from the NCR. Clinical characteristics such as BT topography, histological subtype, and malignancy grade (low-grade (LG) or high-grade (HG)) were retrieved from the NCR and assigned using the ICD-O-3 and the World Health Organisation (WHO) grading system for central nervous system tumours [17].

Within SURVAYA, HRQoL was assessed using the Dutch version of the EORTC QLQ-C30 core questionnaire (version 3.0), divided into six scales: Global Health/Quality of Life (QL), Physical Functioning (PF), Role Functioning (RF), Cognitive Functioning (CF), Emotional Functioning (EF), and Social Functioning (SF). These scales are used as dependent variables for the aim of gaining insights into the different dimensions of HRQoL. Established thresholds of clinical importance (TCIs) are applied to estimate the prevalence of clinically relevant functional impairments [18].

A total of 23 self-reported adverse health outcomes were considered and summarised into 4 categories: 3 general symptoms (fatigue, pain, sleep disturbance), 10 medical conditions (hearing impairments, vision impairments, speech/taste/smell impairments, urinary tract issues, hormonal system issues, cardio-/cerebrovascular system issues, respiratory system issues, digestive system issues, cancer recurrence or metastasis, second primary cancer), 5 neurocognitive symptoms (concentration issues, memory difficulties, brain fog, slowed information processing, multitasking issues), and 5 psychological distress items (fear of cancer recurrence, future uncertainty, depression, body image dissatisfaction, health-related worries). Within SURVAYA, information on adverse health outcomes was assessed either using a dichotomous response option (medical conditions and depression) or a 4-point Likert scale if assessed according to the EORTC measures (all others). Considering the limited sample size, items are dichotomised and used as binary independent variables to streamline further analyses, if adequate. Established TCIs for the EORTC items fatigue, pain, and insomnia were applied, while a cut-off value of TCI = 2 was assumed for other adverse health outcomes. Accordingly, these adverse health outcomes were dichotomised into none-to-mild and moderate-to-severe for further statistical analyses [19].

### Statistical analysis

For comparative analyses, the other 3877 SURVAYA participants (established including all other diagnoses than BT; AYAC) functioned as a control group to determine AYA-BT-specific adverse health outcomes and HRQoL issues. Sociodemographic and clinical characteristics as well as information on adverse health outcomes and HRQoL are summarised using descriptive statistics. Further, they are compared between AYA-BT and AYAC survivors using *χ*^2^-statistics or *t*-tests, as appropriate (*α* = 0.05) and complemented by non-parametric procedures if indicated. With regard to adverse health outcomes, odds ratios (ORs) are estimated for significant associations. Only adverse health outcomes with a prevalence of ≥ 10% are included in further analysis, and subsequent statistical analyses are performed for the AYA-BT population only [20].

To explore the association of adverse health outcomes and HRQoL, six models are employed. Bivariate relationships of the HRQoL dimensions and each adverse health outcome are assessed by univariable models, and adverse health outcomes shown to be significantly associated with the HRQoL dimension (*p* < 0.1) are then included in multivariable analysis. A stepwise backward elimination method utilising bias-corrected AIC is applied to reach final parsimonious models. To maintain the interpretability, a set of fixed control variables consisting of age, sex, education, occupational status, and comorbidity status is forced into the models along with time since diagnosis to account for the cross-cohort design of the SURVAYA study. For assessing the magnitude of clinical relevance, clinically important differences (CIDs) in HRQoL are assessed using evidence-based guidelines [21]. Adjusted mean differences as indicated by the regression coefficients are classified into trivial, small, medium, or large differences, excluding the EF scale due to a lack of established CIDs. All statistical analyses are conducted using Stata 15.1.

## Results

### Sociodemographic and clinical characteristics

Age at study participation ranged from 26 to 57 years in the AYA-BT population while the mean age was significantly lower compared to the AYAC population (42.41 ± 7.23 vs. 45.56 ± 7.46). Sex of the AYA-BT survivors was almost equally distributed, while the majority (61.72%) was female among AYAC survivors. In both populations, more than half of survivors reported to have obtained a university or college degree. A significantly higher unemployment rate was observed in the subpopulation of AYA-BT survivors compared to AYAC survivors (Table 1)*.*

**Table 1:** Sociodemographic and clinical characteristics

		AYA-BT population (*n* = 133)		AYAC population (*n* = 3877)			*p*-value^3^
		*n*	(%)	*n*		(%)	
I. Sociodemographic characteristics							
Age at study participation							
Mean (SD)		42.41 (7.23)		45.56 (7.46)			0.0011*
Range		26–57		23–61			
Median (IQR)		44 (37–48)		45 (40–50)			
< 35 years		22	(16.54)	452		(11.66)	
35–39 years		27	(20.3)	589		(15.19)	
40–44 years		28	(21.05)	881		(22.72)	
45–49 years		38	(28.57)	971		(25.05)	
≥ 50 years		18	(13.53)	984		(25.38)	
Sex							
Male		65	(48.87)	1484		(38.28)	0.014*
Female		68	(51.13)	2393		(61.72)	
Civil status							
Married/registered partnership		72	(54.14)	2143		(55.27)	0.003*
Relationship		26	(19.55)	1092		(28.17)	
Single		35	(26.32)	626		(16.15)	
Missing		0	(0)	16		(0.41)	
Living situation							
Alone		25	(18.8)	468		(12.07)	0.021*
Not alone		108	(81.2)	3404		(87.8)	
Missing		0	(0)	5		(0.13)	
Highest level of education							
Low (no or primary school education)		1	(0.75)	27		(0.7)	0.714^4^
Medium (secondary education)		60	(45.11)	1662		(42.87)	
High (college or university degree)		72	(54.14)	2180		(56.23)	
Missing		0	(0)	8		(0.21)	
Employment status							
Employed		67	(50.38)	2741		(70.7)	< 0.001*
Self-employed		10	(7.52)	547		(14.11)	
Unemployed		56	(42.11)	582		(15.01)	
Missing		0	(0)	7		(0.18)	
Gross monthly income (individual)							
No income		7	(5.26)	99		(2.55)	0.006*
≤ 1500€		21	(15.79)	557		(14.37)	
1501–4000€		59	(44.36)	1628		(41.99)	
> 4000€		17	(12.78)	983		(25.35)	
Missing		29	(21.8)	610		(15.73)	
II. Clinical characteristics							
Age at cancer diagnosis							
Mean (SD)		30.91 (5.65)		31.6 (5.9)			0.183
Range		18–39		18–39			
Median (IQR)		32 (27–35)		33 (28–37)			
18–24 years		20	(15.04)	593		(15.3)	
25–39 years		113	(84.96)	3284		(84.7)	
Time since cancer diagnosis (between diagnosis and survey, in years)							
Mean (SD)		10.97 (4.23)		12.45 (4.51)			< 0.001*
Range		4.95–20.81		4.94–20.85			
Median (IQR)		9.89 (7.66–13.71)		12.42 (8.61–16.03)			
5–10 years		67	(50.38)	1319		(34.02)	
> 10 years		66	(49.62)	2558		(65.98)	
Comorbidities^1^							
No		62	(46.62)	1890		(48.75)	0.495
Yes (1)		36	(27.07)	1066		(27.5)	
Yes (2 +)		29	(21.8)	679		(17.51)	
Missing		6	(4.51)	242		(6.24)	
Primary treatment modality							
Surgery	Yes	128	(96.24)	2998	(77.33)		< 0.001*
	No	5	(3.76)	875	(22.57)		
	Missing	0	(0)	4	(0.10)		
Radiotherapy	Yes	68	(51.13)	1834	(47.30)		0.391
	No	65	(48.87)	2039	(52.59)		
	Missing	0	0	4	(0.10)		
Chemotherapy	Yes	28	(21.05)	2211	(57.03)		< 0.001*
	No	105	(78.95)	1662	(42.87)		
	Missing	0	(0)	4	(0.10)		
Targeted therapy	Yes	1	(0.75)	307	(7.92)		< 0.001*^4^
	No	132	(99.25)	3566	(91.98)		
	Missing	0	(0)	4	(0.10)		
Hormone therapy	Yes	0	(0)	484	(12.48)		< 0.001*^4^
	No	133	(100)	3389	(87.41)		
	Missing	0	(0)	4	(0.10)		
Stem cell therapy	Yes	0	(0)	142	(3.66)		0.015*^4^
	No	133	(100)	3731	(96.23)		
	Missing	0	(0)	4	(0.10)		
BT topography (ICD-O-3 BT sublocalisation)							
Cerebrum (0)		3	(2.26)	**-**			
Frontal lobe (1)		54	(40.6)	**-**			
Temporal lobe (2)		24	(18.05)	**-**			
Parietal lobe (3)		11	(8.27)	**-**			
Ventricle, not otherwise specified (5)		2	(1.5)	**-**			
Cerebellum, not otherwise specified (6)		17	(12.78)	**-**			
Brain stem (7)		7	(5.26)	**-**			
Overlapping lesion of brain (8)		11	(8.27)	**-**			
Brain, NOS (9)		4	(3.01)	**-**			
BT morphology (ICD-O-3)^2^							
Diffuse astrocytoma (9400, LG)		39	(29.32)	**-**			
Oligodendroglioma (9450, LG)		16	(12.03)	**-**			
Anaplastic oligoastrocytoma (9382, HG)		12	(9.02)	**-**			
Anaplastic astrocytoma (9401, HG)		11	(8.27)	**-**			
Ependymoma, NOS (9391, HG)		9	(6.77)	**-**			
Glioblastoma, NOS (9440, HG)		9	(6.77)	**-**			
Medulloblastoma (9470, HG)		9	(6.77)	**-**			
Oligodendroglioma (9451, HG)		7	(5.26)	**-**			
Desmoplastic nodular medulloblastoma (9471, missing)		5	(3.76)	**-**			
Other LG tumour		7	(5.26)	**-**			
Other HG tumour		8	(6.02)	**-**			
Other w/o WHO grade		1	(0.75)				
WHO grading system for central nervous system tumours (low- and high-grade BT)							
LG BT (WHO grade I/II)		62	(46.62)	**-**			
HG BT (WHO grade III/IV)		65	(48.87)	**-**			
Missing		6	(4.51)	**-**			

*LG* low-grade, *HG* high-grade (dichotomised WHO grade in parenthesis), *SD* standard deviation, *IQR* interquartile range

^1^Categorised amount of comorbidities (e.g. anaemia, back pain, rheumatism) based on self-reported data. Missing: not at least *n* = 1 question regarding comorbidities was answered with yes or no

^2^Tumour behaviour of all tumours was/3 malignant, primary

^3^Statistically significant *p* < 0.05

^4^Based on Fisher’s exact-test

Most AYA-BT survivors were diagnosed between the ages of 25 and 39, which was comparable to AYAC survivors. The average time between diagnosis and survey participation was significantly shorter for AYA-BT survivors compared to AYAC survivors (10.97 ± 4.23 vs. 12.45 ± 4.51 years). Approximately half of the AYA-BT survivors participated in the SURVAYA study 5–10 years post-diagnosis, while more of the AYAC survivors participated > 10 years after their diagnosis. Almost all AYA-BT survivors have undergone surgery as part of their cancer therapy. Additionally, more than half of AYA-BT survivors underwent radiotherapy, and approximately a fifth received chemotherapy.

### Adverse health outcomes

While the prevalence of fatigue was significantly higher among AYA-BT survivors, major differences were not found for other general symptoms (Supplement 1). Medical conditions varied in prevalence between the two survivor populations, with AYA-BT survivors experiencing higher rates of certain conditions such as vision impairments and cancer recurrence or metastasis, while they were less affected by urinary tract, hormonal system, and cardio- or cerebrovascular system issues. The prevalence of moderate-to-severe neurocognitive symptoms differed considerably between AYA-BT survivors and AYAC survivors, as the prevalence of all symptoms investigated was higher for AYA-BT survivors with most differences being statistically significant. Psychological distress was comparable between the two populations, except for moderate-to-severe future uncertainty, which was 27.4% more prevalent among AYA-BT survivors.

### Differences in health-related quality of life

Mean values for QL and PF were comparable between AYA-BT survivors and AYAC survivors (Table 2). For the remaining functioning dimensions, mean scores among AYA-BT survivors were significantly lower than those of the AYAC survivors, while CF was the dimension with the largest discrepancy.

**Table 2:** Comparison of health-related quality of life dimensions (mean scores) between the BT and AYAC survivor population

EORTC dimension	AYA-BT population				AYAC population				Mean comparison^2^	
	*n* (missing %^1^)	Mean	SE	(SD)	*n* (missing %^1^)	Mean	SE	(SD)	Mean difference [95%-CI]	*p*-value
QL	125 (6.02%)	73.27	1.40	(15.64)	3600 (7.14%)	75.29	0.29	(17.59)	2.02 [−1.11; 5.15]	0.206^(4)^
PF	126 (5.26%)	88.84	1.46	(16.43)	3617 (6.71%)	91.57	0.23	(13.93)	2.73 [−0.20; 5.66]	0.068^(3,4)^
RF	125 (6.02%)	73.60	2.51	(28.02)	3605 (7.02%)	83.56	0.42	(25.23)	9.96 [5.44; 14.48]	0.000*^(4)^
CF	125 (6.02%)	65.87	2.57	(28.78)	3606 (6.99%)	78.31	0.40	(24.19)	12.46 [7.29; 17.60]	0.000*^(3,4)^
EF	125 (6.02%)	74.13	2.08	(23.30)	3609 (6.91%)	79.65	0.34	(20.50)	5.52 [1.34; 9.70]	0.01*^(3,4)^
SF	124 (6.77%)	78.36	2.6	(28.91)	3602 (7.09%)	88.28	0.36	(21.71)	9.92 [4.73; 15.1]	0.000*^(3,4)^

*SE* standard error, *SD* standard deviation, *CI* confidence interval

^1^Missing values refer to the total population (*n* = 133/*n* = 3877)

^2^*p* < 0.05 is considered significant

^3^Additional Welch test (two-sample *t*-test with unequal variances) was performed

^4^Additional Wilcoxon Ranksum/Man-Whitney-*U*-test was performed

Apart from PF, AYA-BT survivors were significantly more often impaired in their functioning to a clinically relevant extent as revealed by applying TCIs (Supplement 2). The highest prevalence of impairments was identified in CF and EF, as 56.0% and 40.0% of the AYA-BT survivors were impaired to a clinically relevant extent in these domains, respectively, while AYAC survivors were impaired to a significantly lower extent.

### Association of adverse health outcomes and HRQoL in AYA-BT survivors

Table 3 presents associations between prevalent adverse health outcomes and HRQoL as obtained by multiple linear regression, adjusted for control variables and after backward elimination (see Supplement 3 for variable pre-selection and Supplement 4 for full models). A total of *n* = 17 exhibited significant associations with at least one dimension of HRQoL, while the majority of adverse health outcomes showed a negative association with HRQoL. A total of *n* = 27 adjusted mean differences were assessed, while most differences (63.0%) in HRQoL were considered small, 22.2% medium, and 14.8% large.

**Table 3:** Association between adverse health outcomes and HRQoL dimensions: Results of the multivariable linear regression after BE

		Global QOL	Functioning				
			Physical	Role	Cognitive	Emotional^1^	Social^2^
General symptoms	Fatigue (ref = ≤ TCI)	− 8.09*** [− 13.68; − 2.51] **Small**	− 5.46** [− 10.09; − 0.83] **Small**	− 10.23** [− 18.93; − 1.53] **Small**	− 7.45* [− 14.88; − 0.01] **Small**		
	Pain (ref = ≤ TCI)		− 5.31* [− 11.59; 0.97] **Small**	− 10.29* [− 20.99; 0.41] **Small**			
	Sleep disturbance (ref = ≤ TCI)					− 8.64** [− 16.89; − 0.4] **NA**	
Medical conditions	Hearing impairment (ref = no)				− 9.55** [− 18.57; − 0.53] **Medium**		
	Speech/taste/smell issues (ref = no)		− 5.46** [− 10.71; − 0.21] **Small**				− 11.75** [− 21.52; − 1.99] **Medium**
	Urinary tract issues (ref = no)					− 11.39** [− 20.91; − 1.87] **NA**	
	Respiratory system issues (ref = no)						16.04*** [5.51; 26.57] **Large**
	Cancer recurrence or metastasis (ref = no)						− 8.04* [− 17.4; 1.31] **Small**
Neurocognitive symptoms	Memory difficulties (ref = none-to-mild)		6.47* [− 0.27; 13.21] **Small**				
	Brain fog (ref = none-to-mild)		− 13.41*** [− 21.72; − 5.1] **Small**	− 16.73** [− 31.10; − 2.36] **Small**	− 17.89*** [− 30.63; − 5.15] **Large**		− 22.28*** [− 37.77; − 6.79] **Large**
	Slowed information processing (ref = none-to-mild)			− 11.76** [− 22.11; − 1.41] **Small**	− 10.64** [− 20.55; − 0.73] **Medium**	− 10.56*** [− 17.63; − 3.5] **NA**	− 12.2** [− 22.58; − 1.82] **Medium**
	Multitasking issues (ref = none-to-mild)		− 6.17* [− 12.41; 0.07] **Small**		− 16.63*** [− 26.30; − 6.95] **Large**		
Psychological Distress	Fear of cancer recurrence (ref = none-to-mild)					− 10.92** [− 19.67; − 2.17] **NA**	
	Future uncertainty (ref = none-to-mild)	− 5.51* [− 11.3; 0.28] **Small**				− 8.16** [− 14.74; − 1.59] **NA**	− 14.66*** [− 23.31; − 6.02] **Medium**
	Depression (ref = no)	− 9.39** [− 17.6; − 1.19] **Small**		− 14.24** [− 26.12; − 2.37] **Small**			
	Body image dissatisfaction (ref = none-to-mild)				− 11.0** [− 20.43; − 1.58] **Medium**	− 14.61*** [− 23.38; − 5.85] **NA**	
	Health-related worries (ref = none-to-mild)	− 8.61** [− 16.39; − 0.84] **Small**				− 15.95*** [− 25.48; − 6.43] **NA**	

Only statistically significant *β* values are presented while a negative *β* value indicates a negative association between a prevalent adverse health outcome and global health or functioning of -*x* points. Only statistically significant (***p < 0.01, **p < 0.05, *p < 0.1) associations between adverse health outcomes and HRQoL dimensions are shown based on the multiple linear regression models and the conducted backward elimination. Regression coefficients were extracted from each final multiple linear regression model after conducting AIC_c_-based backward elimination and are presented with the 95% CI in square brackets. All analyses were adjusted for age at time of the survey (continuous), sex (dichotomous), educational attainment (dichotomous), employment status (dichotomous), time since diagnosis (continuous), and comorbidity status (categorical). Cells are empty if the adverse health outcome was not included in multiple linear regression (as determined by univariable linear regression), omitted in multiple linear regression, excluded through the process of AIC_c_-based backward elimination, or not statistically significantly associated with the HRQoL dimension. Small/medium/large/NA (not applicable) refers to the established CIDs used for determining the clinical importance of mean differences

*Ref* reference category, *TCI t*hreshold of clinical importance

^1^Additionally controlled for malignancy grade as determined by backward elimination

^2^Additionally controlled for chemotherapy as determined by backward elimination

All general symptoms were associated with at least one functioning dimension, while fatigue also demonstrated a negative association with QL. Medical conditions exhibited associations ranging from a small to a large clinically relevant extent with all functioning scales, while significant associations with QL were not observed. At least moderate levels of neurocognitive symptoms demonstrated a negative and clinically relevant impact on AYA-BT survivors’ functioning (small to large), while most symptoms affected multiple functioning dimensions. Among the psychological adverse health outcomes, the presence of moderate-to-severe future uncertainty, depression, and moderate-to-severe health-related worries was identified as predictors of compromised global HRQoL to a small clinically relevant extent, which were, along with fatigue, the only adverse health outcomes associated with global HRQoL. Additionally, psychological distress items were linked to significantly lower functional health, ranging from small to moderate clinical relevance.

## Discussion

Our study found that AYA-BT survivors reported a variety of adverse health outcomes years beyond their cancer diagnosis, which differed significantly from AYAC survivors. AYA-BT survivors were particularly affected by fatigue, medical conditions such as impairments of vision, speech, taste, or smell, cancer recurrence or metastasis, neurocognitive symptoms, and future uncertainty. While QL and PF were comparable, a significantly higher prevalence of clinically important functional impairments was observed among AYA-BT survivors, particularly in the cognitive and emotional domains. Furthermore, the presence of most adverse health outcomes was associated with a negative and multidimensional impact on HRQoL ranging from small to large clinical relevance.

Our findings indicate that fatigue is not only present in early stages of the disease and during treatment of AYA-BT patients but also continues throughout the disease trajectory into long-term survivorship [14, 22, 23]. In the specific population of AYA-BT patients, the high burden of fatigue can be discussed within the context of epilepsy and required anti-seizure medication alongside other explanations including neurocognitive impairments and BT treatment modalities [24]. Since fatigue might be present in symptom clusters with neurocognitive impairment, psychological distress, and comorbid conditions, a clear differentiation from other symptoms is hampered [25, 26]. Linkages between certain symptoms and conditions may be examined in further research using network analysis [27].

In terms of medical conditions, as mentioned before, AYA-BT survivors report a significantly higher prevalence of impairments or changes in speech, taste, and smell, possibly linked to previous harm due to primary tumour, surgery, or radiotherapy [22, 28]. Additionally, higher rates of cancer recurrence and metastasis in AYA-BT survivors are attributed to the infiltrative nature of BTs and incomplete primary tumour resection or low exposure to systemic treatment due to the blood–brain-barrier and resistance to treatment due to the biology of the BT [29]. Despite the anticipated impact of tumour location and neurotoxic effects of brain tumour-specific treatment, our study did not find a statistically significant difference in the reported frequency of vision-related and hearing impairments among AYA brain tumour survivors compared to the AYAC population [10].

In line with the expectations and previous research, neurocognitive symptoms were identified as AYA-BT-specific issues. Given the nature of the disease, this may be attributable to the initial tumour location and brain-directed treatment impacting functional brain areas [30, 31].

The high prevalence of future uncertainty we found in this study is consistent with the results of other BT-specific studies involving patients and survivors [22, 32, 33]. Since the corresponding item in SURVAYA was rather generic, one can assume that high levels of uncertainty among AYA-BT survivors might be associated with the achievement of life plans, long-term health status, and prognosis [34]. As investigated by Burgers et al. among AYAs with uncertain or poor cancer prognosis, including BT, future uncertainty might also be linked to return-to-work concerns [35].

Our study reaffirms the presence of restricted health in long-term survivorship [36, 37]. The phenomenon of comparable and satisfactory QL and PF scores in AYA-BT and AYAC survivors may be attributed to a response shift altering HRQoL perception [38]. Moreover, measuring HRQoL in AYA-BT survivors must consider a potential distortion due to elevated levels of cognitive impairment. The proportion of participants with a clinically important impairment in functioning domains excluding PF was significantly higher in AYA-BT survivors and highest in EF and CF. High levels of impaired CF identified in this study support prior research conducted by Scholtes et al. and Nicol et al. among childhood and adult BT survivors [20, 30]. Our results highlight the need to address this impairment along with neurocognitive symptoms, which are considered multicausal and may be induced by compression or infiltration of brain tissue due to the tumour and/or by the received cancer treatment, especially considering the effects of radiotherapy [1, 30, 31]. However, while perceived CF and associated cognitive difficulties provide valuable insights into survivors’ experience, its subjective nature may be influenced by physical or psychosocial factors, including depression or anxiety, and symptoms such as fatigue [15, 39]. Significant impairments in the EF dimension are consistent with the current evidence. These might be a result of anxiety and depressive symptoms due to psychosocial challenges or cognitive impairment and, moreover, might be a consequence of pathological processes in certain brain areas and neuropsychological dysfunction [13, 15, 19, 34].

We found that the majority of adverse health outcomes were significantly associated with various HRQoL dimensions, potentially magnifying effects when occurring simultaneously [27, 38]. For instance, cognitive issues might overlap, interrelate, and exacerbate with fatigue or psychological distress which healthcare professionals need to take into account in terms of diagnosis and intervention [10, 40].

Our study confirms that fatigue significantly impacts multiple aspects of HRQoL, consistent with prior research [23, 38]. For example, Nicol et al. identified an association of fatigue with poorer self-perceived CF in adult brain tumour survivors, aligning with our findings [30]. Hence, fatigue disrupts daily life and potentially contributes to the high unemployment observed among BT survivors, as it may hinder return to work [40, 41].

The study also highlights the significant and multidimensional impact of neurocognitive symptoms on survivors’ daily functioning, including challenges in maintaining personal relationships or occupational roles [11, 13, 14]. Additionally, perceived cognitive deficits may be linked with other conditions like underlying mood disorders, as evidenced by the significant relationship observed in this study. As neurocognitive issues might be present in a symptom cluster with fatigue and impact HRQoL, there is a need for screening and management strategies in clinical practise. Clinicians should be aware of these mechanisms, particularly in terms of screening for adverse health outcomes [41].

Psychological adverse health outcomes were identified as predictors of compromised global HRQoL, while the impact of depression is well reported among other cancer survivor populations [42, 43]. Moreover, the negative association of future uncertainty and HRQoL is supported by a single-centre study conducted by Umezaki et al. among glioma patients [32]. Generally, the presence of psychological distress was associated with a multifaceted impact on HRQoL and might affect overall well-being and functional health, which echoes prior cancer survivorship research [44]. Overall, the results of our study largely confirm previous research in other age groups and cancer populations, highlighting the burden of adverse health outcomes and reduced HRQoL faced by cancer survivors. Nevertheless, the impact on younger age groups, including AYAs, is suspected to be significantly larger due to the associated particularities of this phase in life.

The prevalence and impact of physical and psychological adverse health outcomes on AYA-BT survivors’ HRQoL highlight the necessity of long-term follow-up and survivorship care to improve outcomes, including additional and expert professional as well as social support. These insights further emphasise the need for tailored survivorship care plans contributing to efficient and high-quality survivorship care. Holistic survivorship care should cover prevention, early detection, and surveillance of long-term and late effects as well as its management utilising supportive care interventions, while further research on AYA-BT survivors specific supportive care needs is indicated [12]. For instance, physical activity, psychoeducational interventions, cognitive behavioural therapy, or pharmacologic treatments might reduce the major burden of fatigue among BT survivors [41]. In clinical practise, special attention in terms of screening should be given to issues that might occur simultaneously or overlap in symptom clusters, such as fatigue, perceived cognitive issues, and psychological distress [10, 41]. Considering the wide range of long-term and late effects among BT survivors across different medical professions, the need for multidisciplinary survivorship care covering physical and psychosocial issues has been highlighted. Moreover, apart from clinical care, the integral role of family caregivers in supporting AYA-BT survivors and managing adverse outcomes has to be recognised [12, 45]. Next to objective measures, patient-reported outcomes measures (PROMs) serve as supplementary instrument in survivorship care, aiding in the detection of cancer- and treatment-related effects and symptoms, aligning with a patient-centred approach. It is crucial to integrate long-term survivorship and AYA-specific PROMs and HRQoL instruments, including multidimensional PRO instruments for assessing cognitive issues as well as BT-specific items.

## Limitations

To the best of our knowledge, this is the first study to investigate the association of physical and psychological adverse health outcomes and HRQoL in long-term AYA-BT survivors. However, potential sources of bias and limited generalisability must be considered.

First, its cross-sectional design restricts understanding of changes in HRQoL, onset, and trajectories in symptom perception across the time since diagnosis, and only statistically associations were revealed. Further, the small sample size of participants together with the heterogeneity of the populations led to methodological restrictions, emphasising the explorative nature of our analyses. Regarding the sample, it should be noted that it reflects a highly selected subgroup of AYA-BT patients surviving > 5 years. Further, individuals with severe cognitive impairment due to the disease and its treatment did not participate and hence were not considered in our study. Thus, we lack information on non-responders. Additional limitations include the lack of comparisons with the general population regarding HRQoL and adverse health outcomes, as well as the absence of data on specific symptoms relevant to BT survivors, such as motor dysfunction. Moreover, the QLQ-C30 used within SURVAYA is partly too generic to measure all issues of relevance in AYA-BT survivors. Lastly, concerns over the reliability of self-reported data can be raised. However, using validated and multidimensional PROMs alongside objective measures to examine the critical issue of (neuro-)cognitive impairments is reliable for comprehensive assessment.

## Conclusion

This study examined physical and psychological adverse health outcomes in long-term AYA-BT survivors, shedding light on their HRQoL and the impact of adverse health outcomes on various dimensions of HRQoL. Findings reveal that AYA-BT survivors face significant burdens such as fatigue, a variety of medical conditions, neurocognitive issues, and future uncertainty, with most adverse health outcomes negatively impacting HRQoL. This study highlights the need for a comprehensive and multidisciplinary approach to survivorship care, including prevention, surveillance, and management of adverse health outcomes to enhance the quality of AYA-BT survivorship.

## Supplementary Information

Below is the link to the electronic supplementary material.

Supplementary file1 (DOCX 70 KB)

## Data Availability

No datasets were generated or analysed during the current study.

## Abbreviations

AYA: Adolescent and young adult
AYAC: Other AYA cancer
BT: Malignant brain tumour
CID: Clinically important difference
CF: Cognitive functioning
EF: Emotional functioning
HG: High-grade
HRQoL: Health-related quality of life
LG: Low-grade
NCR: Netherlands Cancer Registry
NKI: Netherlands Cancer Institute
OR: Odds ratio
PF: Physical functioning
PRO: Patient-reported outcome
PROM: Patient-reported outcome measure
QL: Global health/quality of life
RF: Role functioning
SF: Social functioning
TCI: Threshold of clinical importance
WHO: World Health Organisation

## References

[1] Walker D, Bendel A, Stiller C, Indelicato D, Smith S, Murray M et al (2017) Central nervous system tumors. In: Bleyer A, Barr R, Ries L, Whelan J, Ferrari A (eds) Cancer Adolesc. Young Adults, Cham: Springer International Publishing, pp 335–81. 10.1007/978-3-319-33679-4_14

[2] Fan Y, Zhang X, Gao C, Jiang S, Wu H, Liu Z et al (2022) Burden and trends of brain and central nervous system cancer from 1990 to 2019 at the global, regional, and country levels. Arch Public Health 80:209. 10.1186/s13690-022-00965-5 36115969 10.1186/s13690-022-00965-5 PMC9482735

[3] Dyba T, Randi G, Bray F, Martos C, Giusti F, Nicholson N et al (2021) The European cancer burden in 2020: incidence and mortality estimates for 40 countries and 25 major cancers. Eur J Cancer 157:308–347. 10.1016/j.ejca.2021.07.039 34560371 10.1016/j.ejca.2021.07.039 PMC8568058

[4] Trama A, Stark D, Bozovic-Spasojevic I, Gaspar N, Peccatori F, Toss A et al (2023) Cancer burden in adolescents and young adults in Europe. ESMO Open 8:100744. 10.1016/j.esmoop.2022.100744 36753992 10.1016/j.esmoop.2022.100744 PMC10024081

[5] Vlooswijk C, Poll-Franse LVVD, Janssen SHM, Derksen E, Reuvers MJP, Bijlsma R et al (2022) Recruiting adolescent and young adult cancer survivors for patient-reported outcome research: experiences and sample characteristics of the SURVAYA study. Curr Oncol 29:5407–5425. 10.3390/curroncol29080428 36005166 10.3390/curroncol29080428 PMC9406992

[6] Alvarez EM, Force LM, Xu R, Compton K, Lu D, Henrikson HJ et al (2022) The global burden of adolescent and young adult cancer in 2019: a systematic analysis for the global burden of disease study 2019. Lancet Oncol 23:27–52. 10.1016/S1470-2045(21)00581-7 34871551 10.1016/S1470-2045(21)00581-7 PMC8716339

[7] Langbecker D, Yates P (2016) Primary brain tumor patients’ supportive care needs and multidisciplinary rehabilitation, community and psychosocial support services: awareness, referral and utilization. J Neurooncol 127:91–102. 10.1007/s11060-015-2013-9 26643806 10.1007/s11060-015-2013-9

[8] Visser O, Ardanaz E, Botta L, Sant M, Tavilla A, Minicozzi P et al (2015) Survival of adults with primary malignant brain tumours in Europe; results of the EUROCARE-5 study. Eur J Cancer 51:2231–2241. 10.1016/j.ejca.2015.07.032 26421825 10.1016/j.ejca.2015.07.032

[9] Alemany M, Velasco R, Simó M, Bruna J (2021) Late effects of cancer treatment: consequences for long-term brain cancer survivors. Neuro-Oncol Pract 8:18–30. 10.1093/nop/npaa039 10.1093/nop/npaa039 PMC7906268 33664966

[10] Figuracion KCF, Halasz LM, Lam N-Y, Goldberg M, Stuckey J, Failor RA et al (2022) Surveillance of long-term complications after treatment of adult brain tumor survivors—review and evidence-based recommendations. Neuro-Oncol Pract 9:475–486. 10.1093/nop/npac053 10.1093/nop/npac053 PMC9665061 36388419

[11] Janssen SHM, Van Der Graaf WTA, Van Der Meer DJ, Manten-Horst E, Husson O (2021) Adolescent and young adult (AYA) cancer survivorship practices: an overview. Cancers 13:4847. 10.3390/cancers13194847 34638332 10.3390/cancers13194847 PMC8508173

[12] Nicklin E, Velikova G, Glaser A, Kwok-Williams M, Debono M, Sarwar N et al (2022) Long-term unmet supportive care needs of teenage and young adult (TYA) childhood brain tumour survivors and their caregivers: a cross-sectional survey. Support Care Cancer 30:1981–1992. 10.1007/s00520-021-06618-7 34636944 10.1007/s00520-021-06618-7 PMC8795012

[13] Van Der Meer PB, Koekkoek JAF, Dirven L, Taphoorn MJB (2022) Quality of life and brain cancer. In: Kassianos AP (ed) Handb. Qual. Life Cancer, Cham: Springer International Publishing, pp 385–408. 10.1007/978-3-030-84702-9_23

[14] Loughan AR, Allen DH, Baumstarck K, Boyer L, Auquier P, Lanoye A et al (2018) Quality of life in neuro-oncology. Handb. Brain Tumor Chemother. Mol. Ther. Immunother., Elsevier, pp 767–81. 10.1016/B978-0-12-812100-9.00061-9

[15] Frances SM, Velikova G, Klein M, Short SC, Murray L, Wright JM et al (2022) Long-term impact of adult WHO grade II or III gliomas on health-related quality of life: a systematic review. Neuro-Oncol Pract 9:3–17. 10.1093/nop/npab062 10.1093/nop/npab062 PMC8789291 35087674

[16] Vandenbroucke JP, von Elm E, Altman DG, Gøtzsche PC, Mulrow CD, Pocock SJ et al (2007) Strengthening the reporting of observational studies in epidemiology (STROBE): explanation and elaboration. Epidemiology 18:805–835. 10.1097/EDE.0b013e3181577511 18049195 10.1097/EDE.0b013e3181577511

[17] WHO (2013) International classification of diseases for oncology (ICD-O), 3rd ed., 1st revision. World Health Organization, Geneva. https://apps.who.int/iris/handle/10665/96612

[18] Giesinger JM, Loth FLC, Aaronson NK, Arraras JI, Caocci G, Efficace F et al (2020) Thresholds for clinical importance were established to improve interpretation of the EORTC QLQ-C30 in clinical practice and research. J Clin Epidemiol 118:1–8. 10.1016/j.jclinepi.2019.10.003 31639445 10.1016/j.jclinepi.2019.10.003

[19] Rogers JL, Vera E, Acquaye A, Briceno N, Jammula V, King AL et al (2021) Living with a central nervous system (CNS) tumor: findings on long-term survivorship from the NIH Natural History Study. Neuro-Oncol Pract 8:460–474. 10.1093/nop/npab022 10.1093/nop/npab022 PMC8278352 34277024

[20] Scholtes C, Baust K, Weinhold L, Creutzig U, Gnekow A, Hinz A et al (2019) Health status, health-related quality of life, and socioeconomic outcome in childhood brain tumor survivors: a German cohort study. Neuro-Oncol 21:1069–1081. 10.1093/neuonc/noz044 30793186 10.1093/neuonc/noz044 PMC6682214

[21] Cocks K, King MT, Velikova G, Martyn St-James M, Fayers PM, Brown JM (2011) Evidence-based guidelines for determination of sample size and interpretation of the European organisation for the research and treatment of cancer quality of life questionnaire core 30. J Clin Oncol 29:89–96. 10.1200/JCO.2010.28.0107 21098316 10.1200/JCO.2010.28.0107

[22] Rimmer B, Bolnykh I, Dutton L, Lewis J, Burns R, Gallagher P et al (2023) Health-related quality of life in adults with low-grade gliomas: a systematic review. Qual Life Res 32:625–651. 10.1007/s11136-022-03207-x 35931881 10.1007/s11136-022-03207-x PMC9992080

[23] Aprile I, Chiesa S, Padua L, Di Blasi C, Arezzo MF, Valentini V et al (2015) Occurrence and predictors of the fatigue in high-grade glioma patients. Neurol Sci 36:1363–1369. 10.1007/s10072-015-2111-7 25698127 10.1007/s10072-015-2111-7

[24] Avila EK, Tobochnik S, Inati SK, Koekkoek JAF, McKhann GM, Riviello JJ et al (2024) Brain tumor-related epilepsy management: a Society for Neuro-oncology (SNO) consensus review on current management. Neuro-Oncol 26:7–24. 10.1093/neuonc/noad154 37699031 10.1093/neuonc/noad154 PMC10768995

[25] Day J, Yust-Katz S, Cachia D, Wefel J, Tremont Lukats IW, Bulbeck H et al (2022) Interventions for the management of fatigue in adults with a primary brain tumour. Cochrane Database Syst Rev 2022. 10.1002/14651858.CD011376.pub3 10.1002/14651858.CD011376.pub3 PMC9466986 36094728

[26] Armstrong TS, Cron SG, Bolanos EV, Gilbert MR, Kang D-H (2010) Risk factors for fatigue severity in primary brain tumor patients. Cancer 2707–15. 10.1002/cncr.25018 10.1002/cncr.25018 20235192

[27] De Rooij BH, Oerlemans S, Van Deun K, Mols F, De Ligt KM, Husson O et al (2021) Symptom clusters in 1330 survivors of 7 cancer types from the PROFILES registry: a network analysis. Cancer 127:4665–4674. 10.1002/cncr.33852 34387856 10.1002/cncr.33852 PMC9291877

[28] Cohen J, Laing DG, Wilkes FJ, Chan A, Gabriel M, Cohn RJ (2014) Taste and smell dysfunction in childhood cancer survivors. Appetite 75:135–140. 10.1016/j.appet.2014.01.001 24412664 10.1016/j.appet.2014.01.001

[29] Rathi S, Griffith JI, Zhang W, Zhang W, Oh J, Talele S et al (2022) The influence of the blood–brain barrier in the treatment of brain tumours. J Intern Med 292:3–30. 10.1111/joim.13440 35040235 10.1111/joim.13440

[30] Nicol C, Ownsworth T, Cubis L, Nguyen W, Foote M, Pinkham MB (2019) Subjective cognitive functioning and associations with psychological distress in adult brain tumour survivors. J Cancer Surviv 13:653–662. 10.1007/s11764-019-00784-8 31313128 10.1007/s11764-019-00784-8

[31] Solanki C, Sadana D, Arimappamagan A, Rao KVLN, Rajeswaran J, Subbakrishna DK et al (2017) Impairments in quality of life and cognitive functions in long-term survivors of glioblastoma. J Neurosci Rural Pract 08:228–235. 10.4103/0976-3147.203829 10.4103/0976-3147.203829 PMC5402490 28479798

[32] Umezaki S, Shinoda Y, Mukasa A, Tanaka S, Takayanagi S, Oka H et al (2020) Factors associated with health-related quality of life in patients with glioma: impact of symptoms and implications for rehabilitation. Jpn J Clin Oncol 50:990–998. 10.1093/jjco/hyaa068 32484212 10.1093/jjco/hyaa068

[33] Flechl B, Ackerl M, Sax C, Dieckmann K, Crevenna R, Gaiger A et al (2012) Neurocognitive and sociodemographic functioning of glioblastoma long-term survivors. J Neurooncol 109:331–339. 10.1007/s11060-012-0897-1 22644537 10.1007/s11060-012-0897-1

[34] Lovely MP, Stewart-Amidei C, Page M, Mogensen K, Arzbaecher J, Lupica K et al (2013) A new reality: long-term survivorship with a malignant brain tumor. Oncol Nurs Forum 40:267–274. 10.1188/13.ONF.267-274 23615139 10.1188/13.ONF.267-274

[35] Burgers VWG, Van Den Bent MJ, Dirven L, Lalisang RI, Tromp JM, Compter A et al (2022) “Finding my way in a maze while the clock is ticking”: the daily life challenges of adolescents and young adults with an uncertain or poor cancer prognosis. Front Oncol 12:994934. 10.3389/fonc.2022.994934 36457502 10.3389/fonc.2022.994934 PMC9706234

[36] Arndt V, Koch-Gallenkamp L, Jansen L, Bertram H, Eberle A, Holleczek B et al (2017) Quality of life in long-term and very long-term cancer survivors versus population controls in Germany. Acta Oncol 56:190–197. 10.1080/0284186X.2016.1266089 28055266 10.1080/0284186X.2016.1266089

[37] Geue K, Sender A, Schmidt R, Richter D, Hinz A, Schulte T et al (2014) Gender-specific quality of life after cancer in young adulthood: a comparison with the general population. Qual Life Res 23:1377–1386. 10.1007/s11136-013-0559-6 24197479 10.1007/s11136-013-0559-6

[38] De Ligt KM, Heins M, Verloop J, Ezendam NPM, Smorenburg CH, Korevaar JC et al (2019) The impact of health symptoms on health-related quality of life in early-stage breast cancer survivors. Breast Cancer Res Treat 178:703–711. 10.1007/s10549-019-05433-3 31512091 10.1007/s10549-019-05433-3 PMC6817812

[39] Lange M, Joly F, Vardy J, Ahles T, Dubois M, Tron L et al (2019) Cancer-related cognitive impairment: an update on state of the art, detection, and management strategies in cancer survivors. Ann Oncol 30:1925–1940. 10.1093/annonc/mdz410 31617564 10.1093/annonc/mdz410 PMC8109411

[40] Klein G, Jodocy D (2021) Symptoms and symptom management in survivorship patients. In: Rauh S (ed) Surviv. Care Cancer Patients, Cham: Springer International Publishing, pp 145–202. 10.1007/978-3-030-78648-9_10

[41] Weis J (2022) Quality of life and cancer-related fatigue: prevalence, assessment and interventions. In: Kassianos AP (ed) Handb. Qual. Life Cancer, Cham: Springer International Publishing, pp 251–64. 10.1007/978-3-030-84702-9_16

[42] Ljungman L, Remes T, Westin E, Huittinen A, Lönnqvist T, Sirkiä K et al (2022) Health-related quality of life in long-term survivors of childhood brain tumors: a population-based cohort study. Support Care Cancer 30:5157–5166. 10.1007/s00520-022-06905-x 35243538 10.1007/s00520-022-06905-x PMC9046139

[43] Mols F, Husson O, Oudejans M, Vlooswijk C, Horevoorts N, Van De Poll-Franse LV (2018) Reference data of the EORTC QLQ-C30 questionnaire: five consecutive annual assessments of approximately 2000 representative Dutch men and women. Acta Oncol 57:1381–1391. 10.1080/0284186X.2018.1481293 29912607 10.1080/0284186X.2018.1481293

[44] Ekels A, Van De Poll-Franse LV, Posthuma EFM, Kieffer J, Issa DE, Koster A et al (2022) Persistent symptoms of fatigue, neuropathy and role-functioning impairment among indolent non-Hodgkin lymphoma survivors: a longitudinal PROFILES registry study. Br J Haematol 197:590–601. 10.1111/bjh.18139 35365860 10.1111/bjh.18139

[45] Neves MC, Bártolo A, Prins JB, Sales CMD, Monteiro S (2023) Taking care of an adolescent and young adult cancer survivor: a systematic review of the impact of cancer on family caregivers. Int J Environ Res Public Health 20:5488. 10.3390/ijerph20085488 37107768 10.3390/ijerph20085488 PMC10138338

