# Supplementary material for: Adverse health outcomes and health-related quality of life (HRQoL) among long-term adolescent and young adult (AYA) brain tumour survivors: results from the population-based SURVAYA study
Source: Support Care Cancer. 2025 Jan 14;33(2):95. doi: 10.1007/s00520-025-09155-9 (PMC11732903; doi:10.1007/s00520-025-09155-9)
Supplement: Supplementary file 1 — Supplementary file1 (DOCX 70 KB) [file 520_2025_9155_MOESM1_ESM.docx]

# Appendices

| **Supplement 1.** Prevalence of Adverse Health Outcomes (n=23) | | | | | | | |
| --- | --- | --- | --- | --- | --- | --- | --- |
|  |  |  | **AYA-BT population**  **(n=133)** | | **AYAC population**  **(n=3877)** | | **p-value^1^** |
|  |  |  | **n** | **%** | **n** | **%** |  |
| **A) General symptoms (n=3)** | | | | | | | |
|  | Fatigue | Yes | 60 | 45.1% | 1,117 | 1,117 | **0.000*** |
|  |  | No | 66 | 49.6% | 2,495 | 2,495 |  |
|  |  | Missing | 7 | 5.3% | 265 | 265 |  |
|  |  | Prevalence^7^ | **47.6%** | | **30.9%** | | **OR^2^=2.03** |
|  | Pain | Yes | 28 | 21.1% | 956 | 24.7% | 0.291 |
|  |  | No | 98 | 73.7% | 2,661 | 68.6% |  |
|  |  | Missing | 7 | 5.3% | 260 | 6.7% |  |
|  |  | Prevalence | **22.2%** | | **26.4%** | |  |
|  | Sleep disturbance/Insomnia | Yes | 22 | 16.5% | 597 | 15.4% | 0.780 |
|  |  | No | 104 | 78.2% | 3,017 | 77.8% |  |
|  |  | Missing | 7 | 5.3% | 263 | 6.8% |  |
|  |  | Prevalence | **17.5%** | | **16.5%** | |  |
| **B) Medical conditions (n=10)** | | | | | | | |
|  | Hearing impairments | Yes | 25 | 18.8% | 517 | 13.3% | 0.082 |
|  |  | No | 102 | 76.7% | 3128 | 80.7% |  |
|  |  | Missing | 6 | 4.5% | 232 | 6.0% |  |
|  |  | Prevalence (%)^7^ | **19.7%** | | **14.2%** | |  |
|  | Reduction in vision/deteriorated vision/eye problem | Yes | 44 | 33.1% | 1221 | 31.5% | 0.838 |
|  |  | No | 84 | 63.2% | 2423 | 62.5% |  |
|  |  | Missing | 5 | 3.8% | 233 | 6.0% |  |
|  |  | Prevalence (%) | **34.4%** | | **33.5%** | |  |
|  | Speech/taste/smell problems | Yes | 33 | 24.8% | 483 | 12.5% | **0.000*** |
|  |  | No | 93 | 69.9% | 3160 | 81.5% |  |
|  |  | Missing | 7 | 5.3% | 234 | 6.0% |  |
|  |  | Prevalence (%) | **26.2%** | | **13.3%** | | **OR=2.32** |
|  | Urinary tract issues | Yes | 18 | 13.5% | 821 | 21.2% | **0.031*** |
|  |  | No | 107 | 80.5% | 2814 | 72.6% |  |
|  |  | Missing | 8 | 6.0% | 242 | 6.2% |  |
|  |  | Prevalence (%) | **14.4%** | | **22.6%** | | **OR=0.58** |
|  | Hormonal system issues | Yes | 15 | 11.3% | 905 | 23.3% | **0.001*** |
|  |  | No | 111 | 83.5% | 2735 | 70.5% |  |
|  |  | Missing | 7 | 5.3% | 237 | 6.1% |  |
|  |  | Prevalence (%) | **11.9%** | | **24.9%** | | **OR=0.41** |
|  | Cardio-/Cerebrovascular system issues | Yes | 10 | 7.5% | 621 | 16.0% | **0.006*** |
|  |  | No | 117 | 88.0% | 3019 | 77.9% |  |
|  |  | Missing | 6 | 4.5% | 237 | 6.1% |  |
|  |  | Prevalence (%) | **7.9%** | | **17.1%** | | **OR=0.42** |
|  | Respiratory system issues | Yes | 26 | 19.5% | 974 | 25.1% | 0.126 |
|  |  | No | 100 | 75.2% | 2667 | 68.8% |  |
|  |  | Missing | 7 | 5.3% | 236 | 6.1% |  |
|  |  | Prevalence (%) | **20.6%** | | **26.8%** | |  |
|  | Digestive system issues | Yes | 24 | 18.0% | 948 | 24.5% | 0.077 |
|  |  | No | 102 | 76.7% | 2691 | 69.4% |  |
|  |  | Missing | 7 | 5.3% | 238 | 6.1% |  |
|  |  | Prevalence (%) | **19.1%** | | **26.1%** | |  |
|  | Cancer recurrence or metastasis | Yes | 32 | 24.1% | 413 | 10.7% | **0.000*** |
|  |  | No | 94 | 70.6% | 3198 | 82.5% |  |
|  |  | Missing | 7 | 5.3% | 266 | 6.9% |  |
|  |  | Prevalence (%) | **25.4%** | | **11.4%** | | **OR=2.64** |
|  | Second primary cancer | Yes | 6 | 4.5% | 272 | 7.0% | 0.244 |
|  |  | No | 120 | 90.2% | 3339 | 86.1% |  |
|  |  | Missing | 7 | 5.3% | 266 | 6.86% |  |
|  |  | Prevalence (%) | **4.8%** | | **7.5%** | |  |
| **C) Neurocognitive symptoms (n=5)** | | | | | | | |
|  | Concentration issues | Moderate-to-severe | 30 | 22.6% | 466 | 12.0% | **0.000*** |
|  |  | None-to-mild | 95 | 71.4% | 3,140 | 81.0% |  |
|  |  | Missing | 8 | 6.0% | 271 | 7.0% |  |
|  |  | Prevalence (%) | **24.0%** | | **12.9%** | | **OR=2.13** |
|  | Memory difficulties | Moderate-to-severe | 34 | 25.6% | 583 | 15.0% | **0.001*** |
|  |  | None-to-mild | 91 | 68.4% | 3,023 | 78.0% |  |
|  |  | Missing | 8 | 6.0% | 271 | 7.0% |  |
|  |  | Prevalence (%) | **27.2%** | | **16.2%** | | **OR=1.94** |
|  | Brain fog | Moderate-to-severe | 17 | 12.8% | 313 | 8.1% | 0.056 |
|  |  | None-to-mild | 108 | 81.2% | 3,296 | 85.0% |  |
|  |  | Missing | 8 | 6.0% | 268 | 6.9% |  |
|  |  | Prevalence (%) | **13.6%** | | **8.7%** | |  |
|  | Slowed information processing | Moderate-to-severe | 40 | 30.1% | 502 | 12.9% | **0.000*** |
|  |  | None-to-mild | 85 | 63.9% | 3,104 | 80.1% |  |
|  |  | Missing | 8 | 6.0% | 271 | 7.0% |  |
|  |  | Prevalence (%) | **32.0%** | | **13.9%** | | **OR=2.91** |
|  | Multitasking issues | Moderate-to-severe | 42 | 31.6% | 599 | 15.5% | **0.000*** |
|  |  | None-to-mild | 83 | 62.4% | 3,007 | 77.6% |  |
|  |  | Missing | 8 | 6.0% | 271 | 7.0% |  |
|  |  | Prevalence (%) | **33.6%** | | **16.6%** | | **OR=2.54** |
| **D) Psychologial distress (n=5)** | | | | | | | |
|  | Fear of cancer recurrence | Moderate-to-severe | 22 | 16.5% | 434 | 11.2% | 0.062 |
|  |  | None-to-mild | 103 | 77.4% | 3,173 | 81.8% |  |
|  |  | Missing | 8 | 6.0% | 270 | 7.0% |  |
|  |  | Prevalence (%) | **17.6%** | | **12.0%** | |  |
|  | Future uncertainty | Moderate-to-severe | 56 | 42.1% | 1001 | 25.8% | **0.000*** |
|  |  | None-to-mild | 68 | 51.1% | 2,605 | 67.2% |  |
|  |  | Missing | 9 | 6.8% | 271 | 7.0% |  |
|  |  | Prevalence (%) | **45.2%** | | **27.8%** | | **OR=2.15** |
|  | Depression | Yes | 17 | 12.8% | 576 | 14.9% | 0.445 |
|  |  | No | 110 | 82.7% | 3,059 | 78.9% |  |
|  |  | Missing | 6 | 4.5% | 242 | 6.2% |  |
|  |  | Prevalence (%) | **13.4%** | | **15.9%** | |  |
|  | Body image dissatisfaction | Moderate-to-severe | 25 | 18.8% | 578 | 14.9% | 0.235 |
|  |  | None-to-mild | 100 | 75.2% | 3,030 | 78.2% |  |
|  |  | Missing | 8 | 6.0% | 269 | 6.9% |  |
|  |  | Prevalence (%) | **20.0%** | | **16.0%** | |  |
|  | Health-related worries | Moderate-to-severe | 20 | 15.0% | 502 | 13.0% | 0.509 |
|  |  | None-to-mild | 105 | 79.0% | 3,105 | 80.1% |  |
|  |  | Missing | 8 | 6.0% | 270 | 6.9% |  |
|  |  | Prevalence (%) | **16.0%** | | **13.9%** | |  |
| Yes: indicates symptom score above corresponding TCI/condition was reported to be prevalent, No: indicates symptom score below or similar to corresponding TCI/ condition was reported to be not prevalent, none-to-mild indicates a non-prevalent symptom since ≤assumed TCI; Moderate-to-severe indicates a prevalent symptom/issue since>assumed TCI; Missing: item was not answered or scale was not available due to missing information in at least associated item; ^1^ Statistically significant p<0.05; ^2^ BT population as independent variable with AYAC survivor population as reference category. | | | | | | | |

| Supplement 2 Comparison of EORTC Functioning Domains with Applied TCIs between the BT and the AYAC Survivor Population. | | | | | | |
| --- | --- | --- | --- | --- | --- | --- |
| EORTC QLQ-C30  Functioning dimension with applied TCI^1^ | | **AYA-BT population**  **(n=133)** | | **AYAC population**  **(n=3877)** | | Chi^2^ test |
|  |  | n | %^2^ | n | %^2^ | p-value^3^ |
| Physical  Functioning | <TCI | 30 | 23.81% | 659 | 18.22% | 0.111 |
|  | ≥TCI | 96 | 76.19% | 2,958 | 81.78% |  |
| Role  Functioning | <TCI | 26 | 20.8% | 499 | 13.84% | **0.028*** |
|  | ≥TCI | 99 | 79.2% | 3,106 | 86.16% |  |
| Cognitive  Functioning | <TCI | 70 | 56.0% | 1,348 | 37.38% | **0.000*** |
|  | ≥TCI | 55 | 44.0% | 2,258 | 62.62% |  |
| Emotional  Functioning | <TCI | 50 | 40.0% | 1,039 | 28.79% | **0.007*** |
|  | ≥TCI | 75 | 60.0% | 2,570 | 71.21% |  |
| Social  Functioning | <TCI | 24 | 19.35% | 357 | 9.91% | **0.001*** |
|  | ≥TCI | 100 | 80.65% | 3,245 | 90.09% |  |
| ^1^ TCI=Threshold of clinical importance, applied in accordance with Giesinger et al. (2020). Score below TCI indicates clinically important functional impairment: PF (TCI=83), RF (TCI=58), CF (TCI=75), EF (TCI=71), SF (TCI=58).  ^2^ Percentages refer to the population with information on EORTC functioning scales available.  ^3^ p<0.05 is considered significant. | | | | | | |

| **Supplement 3** Bivariate relationships between adverse health outcomes and HRQoL: Results of the univariable regression analysis. | | | | | | | | | | | | | | | | | | | | | | | | |
| --- | --- | --- | --- | --- | --- | --- | --- | --- | --- | --- | --- | --- | --- | --- | --- | --- | --- | --- | --- | --- | --- | --- | --- | --- |
|  | **Global Quality of Life** | | | | **Physical Functioning** | | | | **Role Functioning** | | | | **Cognitive Functioning** | | | | **Emotional Functioning** | | | | **Social Functioning** | | | |
| **General symptoms** | Coeff. | [95%-CI] | | sig. | Coeff. | [95%-CI] | | sig. | Coeff. | [95%-CI] | | sig. | Coeff. | [95%-CI] | | sig. | Coeff. | [95%-CI] | | sig. | Coeff. | [95%-CI] | | sig. |
| Fatigue | -11.7 | -16.9 | -6.5 | *** | -10.7 | -16.2 | -5.2 | *** | -21.2 | -30.4 | -11.9 | *** | -26.3 | -35.4 | -17.2 | *** | -17.6 | -25.2 | -9.9 | *** | -21.4 | -31.0 | -11.8 | *** |
| Pain | -12.0 | -18.4 | -5.6 | *** | -12.9 | -19.5 | -6.3 | *** | -24.6 | -35.9 | -13.3 | *** | -21.0 | -32.9 | -9.2 | ** | -17.0 | -26.6 | -7.4 | ** | -22.1 | -33.9 | -10.2 | *** |
| Sleep disturbance | -5.7 | -12.9 | 1.5 | ns | -1.9 | -9.6 | 5.8 | ns | -11.2 | -24.1 | 1.8 | * | -12.8 | -26.1 | 0.4 | * | -15.2 | -25.9 | -4.4 | ** | -14.2 | -27.5 | -1.0 | ** |
| **Medical conditions** | Coeff. | [95%-CI] | | sig. | Coeff. | [95%-CI] | | sig. | Coeff. | [95%-CI] | | sig. | Coeff. | [95%-CI] | | sig. | Coeff. | [95%-CI] | | sig. | Coeff. | [95%-CI] | | sig. |
| Hearing impairments | -6.0 | -12.9 | 0.9 | * | -10.6 | -17.3 | -3.9 | *** | -9.6 | -22.0 | 2.8 | ns | -16.2 | -28.6 | -3.7 | ** | -5.4 | -15.8 | 4.9 | ns | 1.4 | -11.5 | 14.3 | ns |
| Eyes/vision impairments | -3.9 | -9.7 | 2.0 | ns | -6.4 | -12.4 | -0.3 | ** | -10.0 | -20.3 | 0.4 | * | -14.1 | -24.6 | -3.7 | *** | -5.8 | -14.4 | 2.9 | ns | -2.5 | -13.3 | 8.4 | ns |
| Speech/taste/smell issues | -6.3 | -12.6 | -0.1 | ** | -10.4 | -16.8 | -4.1 | *** | -15.9 | -26.7 | -5.1 | *** | -13.9 | -25.4 | -2.4 | ** | -14.2 | -23.4 | -5.0 | *** | -17.2 | -28.8 | -5.6 | *** |
| Urinary tract issues | -7.3 | -15.3 | 0.7 | * | -17.5 | -25.3 | -9.7 | *** | -14.5 | -28.5 | -0.5 | ** | -17.2 | -32.2 | -2.1 | ** | -18.8 | -30.5 | -7.1 | *** | -18.3 | -33.5 | -3.1 | ** |
| Hormonal system issues | -0.8 | -9.3 | 7.7 | ns | -5.3 | -14.1 | 3.5 | ns | -4.8 | -19.7 | 10.2 | ns | -5.7 | -21.4 | 10.0 | ns | 1.7 | -11.1 | 14.5 | ns | 3.2 | -12.7 | 19.1 | ns |
| Respiratory system issues | 4.6 | -2.2 | 11.5 | ns | -3.5 | -10.7 | 3.7 | ns | -1.5 | -13.9 | 10.8 | ns | 3.7 | -9.1 | 16.5 | ns | 3.5 | -6.7 | 13.7 | ns | 12.7 | 0.2 | 25.2 | ** |
| Digestive system issues | 0.4 | -6.8 | 7.6 | ns | -4.1 | -11.5 | 3.3 | ns | -11.3 | -24.0 | 1.5 | * | -3.1 | -16.2 | 9.9 | ns | -5.4 | -15.8 | 5.1 | ns | -8.3 | -21.5 | 5.0 | ns |
| Metastasis / recurrence | -0.3 | -6.7 | 6.1 | ns | -2.7 | -9.5 | -3.9 | ns | -4.5 | -15.9 | 6.9 | ns | 4.6 | -7.2 | 16.4 | ns | 1.17 | -8.4 | 10.7 | ns | -10.3 | -22.0 | 1.4 | * |
| **Neurocognitive symptoms** | Coeff. | [95%-CI] | | sig. | Coeff. | [95%-CI] | | sig. | Coeff. | [95%-CI] | | sig. | Coeff. | [95%-CI] | | sig. | Coeff. | [95%-CI] | | sig. | Coeff. | [95%-CI] | | sig. |
| Concentration issues | -10.9 | -17.3 | -4.6 | *** | -14.0 | -20.3 | -7.8 | *** | -30.5 | -40.9 | -20.1 | *** | -^1^ | | | | -20.1 | -29.2 | -11.0 | *** | -23.3 | -34.9 | -11.8 | *** |
| Memory difficulties | -10.8 | -16.8 | -4.8 | *** | -10.4 | -16.6 | -4.2 | *** | -22.3 | -32.8 | -11.7 | *** | -^1^ | | | | -18.7 | -27.5 | -10.0 | *** | -18.6 | -29.7 | -7.4 | *** |
| Brain fog | -16.5 | -24.3 | -8.7 | *** | -18.0 | -25.9 | -10.1 | *** | -37.9 | -51.3 | -24.6 | *** | -51.2 | -63.0 | -39.3 | *** | -31.3 | -42.0 | -20.6 | *** | -34.9 | -49.5 | -20.3 | *** |
| Information processing | -10.2 | -16.0 | -4.5 | *** | -13.6 | -19.4 | -7.8 | *** | -30.1 | -39.5 | -20.8 | *** | -38.5 | -47.2 | -29.8 | *** | -20.2 | -28.3 | -12.1 | *** | -27.3 | -37.3 | -17.2 | *** |
| Multitasking issues | -9.9 | -15.6 | -4.3 | *** | -13.3 | -18.9 | -7.6 | *** | -26.6 | -36.1 | -17.2 | *** | -40.6 | -48.7 | -32.6 | *** | -14.9 | -23.3 | -6.5 | *** | -19.9 | -30.2 | -9.6 | *** |
| **Psychological distress** | Coeff. | [95%-CI] | | sig. | Coeff. | [95%-CI] | | sig. | Coeff. | [95%-CI] | | sig. | Coeff. | [95%-CI] | | sig. | Coeff. | [95%-CI] | | sig. | Coeff. | [95%-CI] | | sig. |
| Fear of cancer recurrence | -3.0 | -10.2 | 4.3 | ns | 1.5 | -6.2 | 9.2 | ns | 0.8 | -12.3 | 13.9 | ns | -1.6 | -15.0 | 11.9 | ns | -21.0 | -31.1 | -10.9 | *** | -7.8 | -21.2 | 5.7 | ns |
| Future uncertainty | -10.8 | -16.1 | -5.5 | *** | -8.1 | -13.8 | -2.3 | *** | -19.0 | -28.5 | -9.5 | *** | -19.5 | -29.1 | -9.9 | *** | -19.0 | -26.4 | -11.6 | *** | -23.0 | -32.5 | -13.4 | *** |
| Depression | -12.7 | -20.5 | -5.0 | *** | -7.0 | -15.5 | 1.4 | ns | -30.7 | -44.2 | -17.3 | *** | -15.0 | -29.6 | -0.3 | ** | -^1^ | | | | -13.6 | -28.4 | 1.3 | * |
| Body image dissatisfaction | -8.7 | -15.6 | -1.8 | ** | -11.6 | -18.6 | -4.6 | *** | -23.1 | -35.1 | -11.1 | *** | -33.4 | -44.8 | -22.0 | *** | -24.1 | -33.3 | -14.9 | *** | -22.3 | -34.7 | -9.8 | *** |
| Health-related worries | -14.8 | -21.9 | -7.7 | *** | -9.3 | -17.1 | -1.5 | ** | -24.1 | -37.0 | -11.2 | *** | -19.4 | -32.9 | -5.8 | *** | -33.1 | -42.7 | -23.6 | *** | -26.2 | -39.8 | -12.7 | *** |
| Results obtained by univariable linear regression analysee; Dependent variable: EORTC dimension; Independent variable: condition/symptom (reference=non-prevalent condition/symptom)  *** p<0.01, ** p<0.05, * p<0.1; ns=not significant.  ^1^ omitted, part of EORTC subscale. | | | | | | | | | | | | | | | | | | | | | | | | |

| Supplement 4 Association between adverse health outcomes and HRQoL dimensions: Results of the multivariable linear regression after backward elimination (full models). | | | | | | | |
| --- | --- | --- | --- | --- | --- | --- | --- |
|  | | **Global QOL** | **Functioning** | | | | |
|  |  |  | **Physical** | **Role** | **Cognitive** | **Emotional** | **Social** |
| Sociodemographic and clinical  control variables | Age at survey^1^ | -0.03 (0.24) | -0.08 (0.2) | 0.45 (0.36) | 0.24 (0.31) | -0.34 (0.28) | 0.29 (0.38) |
|  | Female (ref=male)^1^ | 4.18 (2.71) | -3.16 (2.3) | 2.19 (4.08) | 1.57 (3.53) | **9.88***** (3.2) | 4.39 (4.29) |
|  | No higher edu. (ref=higher edu.)^1^ | **-5.2*** (2.68) | **-6.06***** (2.29) | 1.43 (4.05) | **-6.67** (3.43)** | -2.54 (3.12) | -1.56 (4.27) |
|  | Unemployed (ref=employed)^1^ | -3.43 (3.05) | -3.84 (2.85) | -**10.43**** (4.78**)** | -6.54 (4.17) | 2.71 (3.62) | -5.33 (4.89) |
|  | No partner (ref=yes) | x | x | x | x | x | x |
|  | Time since diagnosis^1^ | -0.05 (0.39) | -0.36 (0.33) | -0.46 (0.59) | 0.32 (0.51) | 0.3 (0.47) | 0.09 (0.63) |
|  | Chemotherapy | x | x | x | x | x | 8.49 (5.21) |
|  | Radiotherapy | x | x | x | x | x | x |
|  | Malignancy grade (ref=LG) | x | x | x | x | **-5.19*** (3.06) | x |
|  | Comorbidity: 1 (ref=none)^1^ | 1.38 (3.25) | -1.34 | -5.83 (4.91) | -4.33 (4.1) | -5.95 (3.75) | **-9.82*** (5.1) |
|  | Comorbidity: 2+ (ref=none)^1^ | -2.5 (3.31) | 2.22 | **-12.76**** (5.02) | -5.53 (4.23) | -5.22 (3.9) | **-18.17***** (5.17) |
| General  symptoms | Fatigue (ref=≤TCI) | **-8.09***** (2.82) | **-5.46**** (2.33) | **-10.23**** (4.39) | **-7.45*** (3.75) | x | x |
|  | Pain (ref=≤TCI) | x | **-5.31*** (3.17) | **-10.29*** (5.39) | x | x | x |
|  | Sleep disturbance (ref=≤TCI) | - | - | x | x | **-8.64**** (4.16) | x |
| Medical conditions | Hearing impairment (ref=no) | x | x | x | **-9.55**** (4.55) | - | - |
|  | Eyes/vision impairments (ref=no) | - | x | - | x | **-** | - |
|  | Speech/taste/smell issues (ref=no) | x | **-5.46**** (2.64) | x | x | x | **-11.75**** (4.92) |
|  | Urinary tract issues (ref=no) | x | x | x | x | **-11.39**** (4.8**)** | x |
|  | Hormonal system issues (ref=no) | - | - | - | - | - | - |
|  | Respiratory system issues (ref=no) | - | - | - | - | - | **16.04***** (5.31) |
|  | Digestive system issues (ref=no) | - | - | x | - | - | - |
|  | Cancer recurrence/metastasis (ref=no) | - | - | - | - | - | **-8.04*** (4.72) |
| Neurocognitive symptoms | Concentration issues (ref=none-to-mild) | x | **x** | x | -^2^ | x | x |
|  | Memory difficulties (ref=none-to-mild) | x | **6.47*** (3.4) | x | -^2^ | x | x |
|  | Brain fog (ref=none-to-mild) | x | **-13.41***** (4.19) | **-16.73**** (7.24) | **-17.89***** (6.42) | x | **-22.28***** (7.81) |
|  | Slowed information processing (ref=none-to-mild) | x | x | **-11.76**** (5.22) | **-10.64**** (5.0) | **-10.56***** (3.56) | **-12.2**** (5.23) |
|  | Multitasking issues (ref=none-to-mild) | x | **-6.17*** (3.15) | x | **-16.63***** (4.88) | x | x |
| Psychological Distress | Fear of cancer recurrence (ref=none-to-mild) | - | - | - | - | **-10.92**** (4.41) | - |
|  | Future uncertainty (ref=none-to-mild) | **-5.51*** (2.92) | x | x | x | **-8.16**** (3.31) | **-14.66***** (4.36) |
|  | Depression (ref=no) | **-9.39**** (4.14) | - | **-14.24**** (5.99) | x | -^2^ | x |
|  | Body image dissatisfaction (ref=none-to-mild) | x | x | x | **-11.0**** (4.75) | **-14.61***** (4.42) | x |
|  | Health-related worries (ref=none-to-mild) | **-8.61**** (3.92) | x | x | x | **-15.95***** (4.8) | x |
| β_0_ | **Intercept** | **85.95***** (8.25) | **109.96***** (6.96) | **81.8***** (12.44) | **77.88***** (10.61) | **103.55***** (9.73) | **86.06***** (13.26) |
| Regression statistics | Observations (n) | 114 | 114 | 114 | 114 | 115 | 114 |
|  | Model significance (F test) | F(11, 102)=5.16,  p=0.000 | F(13, 100)=7.87, p=0.000 | F(12, 101)= 8.75, p=0.000 | F(13, 100)= 15.66, p=0.000 | F(15, 99)=9.27, p=0.000 | F(14, 99)=8.31, p=0.000 |
|  | R^2^ | 0.3576 | 0.5058 | 0.5096 | 0.6706 | 0.5842 | 0.5404 |
|  | Adj. R^2^ | **0.2883** | **0.4415** | **0.4513** | **0.6278** | **0.5212** | **0.4754** |
| Ref=reference category; TCI=Threshold of Clinical Importance; p-values of the corresponding regression coefficient (*** p<0.01 ** p<0.05, * p<0.1); regression coefficients were extracted from each final multiple linear regression model after conducting AIC_c_-based backward elimination; Standard error in parentheses.  -: Adverse health outcome was not included in multiple linear regression (as determined by univariable linear regression or omitted)  x: excluded through the process of AIC_c_-based backward elimination.  ^1^ fixed set of control variables (not affected by AIC_c_-based backward elimination)  ^2^ omitted (part of EORTC subscale) | | | | | | | |
